# Pathogenesis and Immune Response of Ebinur Lake Virus: A Newly Identified *Orthobunyavirus* That Exhibited Strong Virulence in Mice

**DOI:** 10.3389/fmicb.2020.625661

**Published:** 2021-02-01

**Authors:** Lu Zhao, Huanle Luo, Doudou Huang, Ping Yu, Qiannan Dong, Caroline Mwaliko, Evans Atoni, Raphael Nyaruaba, Jiangling Yuan, Guilin Zhang, Dennis Bente, Zhiming Yuan, Han Xia

**Affiliations:** ^1^Key Laboratory of Special Pathogens and Biosafety, Center for Biosafety Mega-Science, Wuhan Institute of Virology, Chinese Academy of Sciences, Wuhan, China; ^2^University of Chinese Academy of Sciences, Beijing, China; ^3^School of Public Health (Shenzhen), Sun Yat-sen University, Guangzhou, China; ^4^Computing Virus Discipline Group, Wuhan Institute of Virology, Chinese Academy of Sciences, Wuhan, China; ^5^The Center for Disease Control and Prevention of Xinjiang Uygur Autonomous Region, Urumqi, China; ^6^Xinjiang Heribase Biotechnology Co., Ltd., Urumqi, China; ^7^Department of Microbiology & Immunology, University of Texas Medical Branch, Galveston, TX, United States

**Keywords:** Ebinur Lake virus, Orthobunyavirus, pathogenesis, immune response, BALB/c mouse

## Abstract

*Orthobunyaviruses* are a group of viruses with significant public and veterinary health importance. These viruses are mainly transmitted through mosquito-, midge-, and tick-vectors, and are endemic to various regions of the world. Ebinur Lake virus (EBIV), a newly identified member of *Orthobunyavirus*, was isolated from *Culex* mosquitoes in Northwest China. In the present study, we aimed to characterize the pathogenesis and host immune responses of EBIV in BALB/c mice, as an animal model. Herein, we determined that BALB/c mice are highly susceptible to EBIV infection. The infected mice exhibited evident clinical signs including weight loss, mild encephalitis, and death. High mortality of mice was observed even with inoculation of one plaque-forming unit (PFU) of EBIV, and the infected mice succumbed to death within 5–9 days. After EBIV challenge, rapid viremic dissemination was detected in the peripheral tissues and the central nervous system, with prominent histopathologic changes observed in liver, spleen, thymus, and brain. Blood constituents’ analysis of EBIV infected mice exhibited leukopenia, thrombocytopenia, and significantly elevated ALT, LDH-L, and CK. Further, EBIV infection induced obvious cytokines changes in serum, spleen, and brain in mice. Collectively, our data describe the first study that systematically examines the pathogenesis of EBIV and induced immune response in an immunocompetent standard mouse model, expanding our knowledge of this virus, which may pose a threat to One Health.

## Introduction

Mosquitoes are known to act as reservoirs of extensive pathogens (Xia et al., 2018; Nyaruaba et al., 2019), several mosquito-borne viruses (arboviruses) causing human diseases remain the global public health concerns (Tandina et al., 2018). However, with the constant evolution and crossing species barrier, the emergence of novel zoonotic pathogens is one of the greatest challenges to global health security (Borland et al., 2020). Cooperation among human, animal, and environmental sciences to combat emerging public health threats has become an important issue under the One Health Initiative (Ryu et al., 2017). Therefore, these potential novel zoonotic pathogens still should not be neglected (Cunningham et al., 2017). Thus, it is critical to identify novel pathogens with zoonotic potential to reduce their risk of emergence using surveillance programs (Scarpino et al., 2017; Ramírez et al., 2018). Over the past decade, there have been key scientific advances in arbovirus surveillance and have sped up the discovery of these viruses (Shi et al., 2016; Halbach et al., 2017). Ebinur Lake virus (EBIV) was discovered and isolated from *Culex modestus* mosquitoes through a surveillance study done in the Ebinur Lake region in China, 2014, which was identified as a novel member of the genus *Orthobunyavirus* within the family *Peribunyaviridae*, Bunyamwera serogroup.

*Orthobunyavirus* is the largest and most diverse genus of bunyaviruses, comprising of more than 170 viruses divided into more than 20 serogroups based on serological relatedness of complement-fixing antibodies (mediated by N protein), hemagglutinating and neutralizing antibodies (mediated by glycoproteins; Shchetinin et al., 2015; Palya et al., 2019). The main hosts of these viruses include rodents, primates, birds, ungulates, and humans (Calisher, 1996), and some members have been reported to cause disease in these vertebrate hosts. Bunyamwera virus (BUNV) and Cache Valley virus (CVV) can cause severe symptoms in ruminants, such as spontaneous abortion and teratogenic effects (Rodrigues Hoffmann et al., 2013), which have caused a considerable economic loss in the livestock industry (Kim et al., 2011). Besides, BUNV was also proved to infect free ranging birds (Tauro et al., 2009). In humans, BUNV, Germiston virus (GERV), Ilesha virus (ILEV), Fort Sherman virus (FSV), and Guaroa virus (GROV) are known to cause disease with symptoms such as febrile illness (Schwartz and Allen, 1970; Aguilar et al., 2010; Pachler et al., 2013; Dutuze et al., 2018; de Oliveira Filho et al., 2020), and encephalitis caused by Tensaw virus (TENV; Calisher et al., 1988). Thus, Bunyamwera serogroup viruses are a cause for concern in public and veterinary health (Rogers et al., 2017; Dutuze et al., 2018). Some newly discovered members in this group have been reported with potential infection risk in humans and/or animals, but they are not yet well characterized (Sudeep et al., 2018). Therefore, except for the epidemiological studies, pathogenesis studies are critically needed to identify and understand disease threats to humans, livestock, and wildlife.

In experimental animal models, *Orthobunyaviruses* could cause neurological diseases that involve neuroinvasive disease and neurovirulent disease as seen in the members of California serogroup (CSG). This can be evaluated by several parameters such as the route of inoculation. Neuroinvasion represents virus spread to the central nervous system (CNS) following peripheral inoculation [intraperitoneally, (i.p.)], while neurovirulence describes the lethal infection following direct route of inoculation [intracranial, (i.c.) or intranasal, (i.n.)] with the virus. The majority of pathogenesis studies of the CSG viruses have focused on La Crosse virus (LACV). In LACV-infected mice, short viremia was observed and the viral antigen was not detected in peripheral tissues, but in neurons. GERV presumably circumvented the normal killing mechanisms of macrophages and replicated in these cells in mice (Olson et al., 1975). Since research is insufficient, more studies on *Orthobunyaviruses* need to be carried out to examine the differences in mechanistic pathogenesis (Evans and Peterson, 2019).

In our previous report, EBIV was found to replicate efficiently and form cytopathic effect (CPE) in different vertebrate cells. The preliminary data also demonstrated that EBIV is able to cause lethal disease and pathological changes in Kunming mice. In addition, the IgM, IgG, and neutralizing antibodies against EBIV have been detected in the residents. Therefore, the further understanding of the detailed pathogenic mechanism and the host immune response to the virus is important in the risk assessment of EBIV infection in animals or humans.

In this study, we fully characterized EBIV infection using a BALB/c mice model, and carried out all aspects of EBIV induced hematology, clinical chemistry, tissue tropism, and immunology changes in hosts for the first time, providing a better understanding of viral pathogenesis and host immune status against EBIV infection, and endorsing the One Health Initiative.

## Materials and Methods

### Ethics Statement

Animal studies were approved by the Laboratory Animal Ethics Committee of Wuhan Institute of Virology, Chinese Academy of Sciences (Approval No. WIVA12201901). All animal procedures were performed in strict compliance with the guidelines of Guide for the Care and Use of Laboratory Animals (National Research Council, 2011).

### Cell Line, Virus Stock, and Animal

Baby hamster kidney, BHK-21 cell line was used in this study. BHK-21 was maintained at 37°C in Dulbecco’s minimal essential medium (DMEM; 4.5 g/liter D-glucose) containing 10% fetal bovine serum (FBS) and 1% penicillin/streptomycin in 5% CO_2_. EBIV isolate Cu-XJ20 was first isolated from *Culex modestus* mosquitoes in Xinjiang, China (Xia et al., 2020). The EBIV virus stock was propagated in BHK-21 cells in DMEM containing 2% FBS and stored in aliquots at −80°C. Adult BALB/c mice (6–8-week-old) were provided by the Animal Centre of Wuhan Institute of Virology. The mice were maintained in the ABSL-2 facility with controlled temperature (22°C), humidity, and a 12-h light/dark cycle.

### Plaque Assay

Virus titrations were performed using BHK-21 cells as previously described (Xia et al., 2020), and the results expressed as PFU ml^−1^.

### Median Lethal Dose Challenge and Pathogenesis Experiments

For the median (50%) lethal dose (LD_50_) experiment, 10 groups of male and female BALB/c mice (*n* ≥ 10 per group) were infected intraperitoneally (i.p.) with EBIV at 10^5^–10^−4^ PFU of 10-fold dilutions per animal in 200 μl serum-free DMEM. Control group/mock mice (*n* ≥ 5) were inoculated with 200 μl serum-free DMEM. These mice were then observed at least once a day, and behavioral and weight changes were monitored over 2 weeks. The experiment was repeated three times independently.

For pathogenesis experiments, only female mice were administered with 10 PFU EBIV in 200 μl serum-free DMEM through the i.p. route. Mock-infected mice received 200 μl serum-free DMEM. From day 1 to 5 post-infection, EBIV-infected mice (*n* = 5) and mock-infected mice (*n* = 3–5) were euthanized by isoflurane overdose. About 200 μl of blood was collected from the orbital sinus by a capillary tube daily for viremia assay. Whole blood was also collected from infected and mock-infected mice for biochemical analysis after removing their eyes. During necropsy, the organs were macroscopically observed, and afterward the tissues (liver, spleen, kidney, intestine, lung, brain, thymus, and Peyer’s patch) harvested were divided into three parts, one for determination of titers, the second one was stored in 15 ml centrifuge tubes containing 10% Paraformaldehyde for histopathological and immunohistochemical (IHC) assays, and the last one was sectioned and stored in 2.5% glutaraldehyde solution for ultrastructural analysis.

### Quantification of Virus in Mice

To determine the viremia, blood samples were first kept at 4°C for 4 h, followed by centrifugation at 3,000 × *g* for 10 min to separate the serum before storage at −80°C until further use. Viremia titers were tittered by plaque assay on BHK-21 cells. For quantification of the virus in tissues, the tissues were first removed and weighed before being homogenized by a T grinder electric tissue grinder OSE-Y30 (TIANGEN, China) on ice using sterile pestles with serum-free DMEM. Supernatants were then collected and stored at −80°C. Finally, the viral titers were determined by plaque assay, and titers were expressed as PFU g^−1^.

### Histopathology and Immunohistochemical Assay

For the histopathological analysis, tissues of EBIV-infected and mock-infected mice fixed with 10% paraformaldehyde were embedded in paraffin, cut, and stained with hematoxylin-eosin (HE), and examined under light microscopy. For the IHC assay, the antiserum against EBIV-NP recombination protein produced in BALB/c mice was diluted at 1:100, and the histological sections were cut to 4–5 μm for immunohistochemical assay as previously described (Sawatsky et al., 2014; Matos et al., 2019). The stained sections were evaluated for EBIV immunoreactivity by Image-Pro Plus 6.0 software (Media Cybernetics, Inc., Silver Spring, MD, United States), and the accumulated optical density (IOD) and the corresponding brown-yellow positive area were provided with three randomly selected fields of view (200×). Finally, each group was represented by the accumulated optical density IOD (SUM; Zhang et al., 2016; Liu et al., 2019). All the above were performed at Wuhan Biotechnology Corporation.

### Ultrastructural Analysis

Ultrathin sections of tissues in EBIV-infected and mock-infected mice were consistent as previously described (Otto et al., 2017) using an FEI Tecnai G20 transmission electron microscope (FEI Company, United States) at 200 kV.

### Hematology and Clinical Chemistry

For complete blood counts, 50 μl of whole blood was collected in an anticoagulant tube. HemaVet 950FS hematology analyzer equipped with software was used to measure the white blood cell (WBC) count, red blood cell (RBC) count, hemoglobin (Hb) concentration, hematocrit, and platelet count. For clinical chemistry, 200 μl of serum was analyzed by VetScan2 Chemistry Analyzer (Abaxis Inc., Sunnyvale, CA, United States), which provides a diagnostic panel, including albumin, total bilirubin, alanine aminotransferase, alkaline phosphatase, glucose, amylase, calcium, urea nitrogen, creatinine, lactate dehydrogenase, and creatine kinase.

### Quantification of Cytokines

To determine cytokine levels in the serum and tissues of mice, 25 μl of serum or tissue homogenate (liver, spleen, and brain) was added to each well (triplicate wells) in the premixed assay panels using a Bio-Plex Pro™ Mouse Cytokine Grp I Panel 8-Plex kit (Bio-Rad) according to the manufacturer’s instructions, then interleukin IL-1β, IL-2, IL-4, IL-5, IL-10, interferon (IFN)-γ, and tumor necrosis factor (TNF)-α of samples were qualified and analyzed by the Bio-Plex 200 System (Bio-Rad) and the Bio-Plex Manager software (version 6.0).

### Statistical Analysis

All statistical analyses were done using R v4.0.2, the comparisons are ensured by unpaired Student’s *t*-test and the LD_50_ is estimated by Probit regression model. Levels of statistical significance are given as either *p* < 0.05 or 0.01.

## Results

### BALB/c Mice Succumbed to EBIV Infection Rapidly

**Figure 1:**
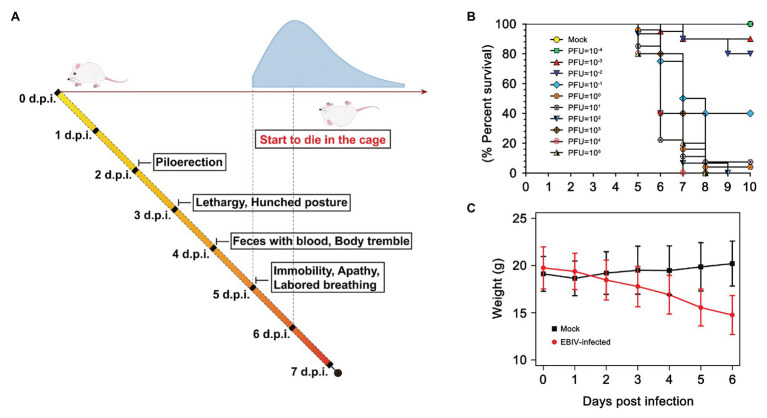
Clinical illness and survival curve during Ebinur Lake virus (EBIV) infection in male and female BALB/c mice. **(A)** Behavioral changes. **(B)** Survival curve. Adult mice (n ≥ 10 per group) were challenged intraperitoneally (i.p) with dose from 10^−4^ to 10^5^ plaque-forming unit (PFU). **(C)** Weight change over 6 days when challenged with dose of 10 PFU.

BALB/c mice exhibited clinical signs of disease from 2 days post-infection (d.p.i), which typically manifested as piloerection, lethargy, and hunched posture (Figure 1A). The weight of EBIV-infected mice also began to decrease from 2 d.p.i until death with a mean weight loss of 19.85% (Figure 1C). Feces with blood and body tremble were observed at 4 d.p.i. From 5 d.p.i, the mice progressively became immobile, weak with labored breathing, and finally started to die, with the peak of mice’s death at 6 d.p.i. The survival curve showed more than 90% of BALB/c mice succumbed to death when administrated with an extremely low dose of 1 PFU EBIV, suggesting that BALB/c mice are highly permissive to EBIV infection. The LD_50_ was calculated as 0.046 PFU by logistic regression (Figure 1B) and 10 PFU was selected as the infectious dose of the following experiments in this study.

### EBIV Disseminates From the Peripheral Tissue to the Central Nervous System

**Figure 2:**
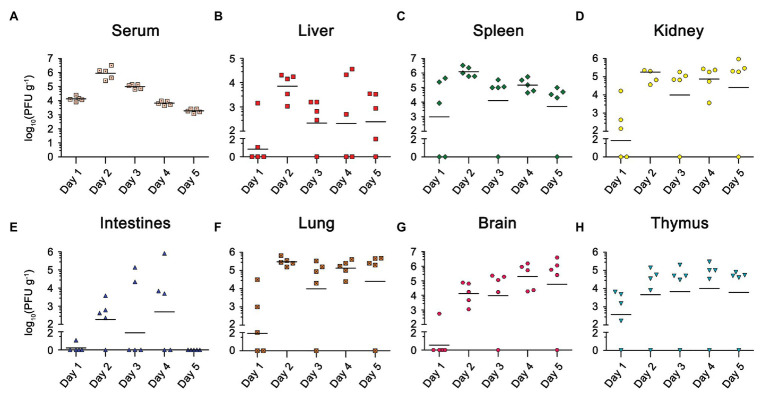
Viral load in different organs of EBIV infected female BALB/c mice inoculated by i.p with 10 PFU. Viral titer was measured and quantified in serum and organs from day 1 to 5 post-infection by plaque assay (*n* = 5). Viral titers for the Serum **(A)**, Liver **(B)**, Spleen **(C)**, Kidney **(D)**, Lung **(E)**, Intestines **(F)**, Brain **(G)**, and Thymus **(H)** are shown.

Viremia was detected in all five mice from 1 d.p.i, peaked at 2 d.p.i, and then continued to decrease (Figure 2A). We also found a high viral load in the lymphoid organs (spleen and thymus) isolated from most of the infected mice at 1 d.p.i, suggesting EBIV mainly disseminated in the blood and lymphoid organs at the early stage (Figures 2C,H). After 2 d.p.i, we detected increased viral particles in the kidney (Figure 2D), lung (Figure 2F), liver (Figure 2B), intestine (Figure 2E), and thymus (Figure 2H). The viral titers in these organs almost remained at the same level until 5 d.p.i. However, the viral load was slightly decreased in the spleen after reaching its peak at 2 d.p.i. In contrast to the spleen, the virus titer in the brain continued to rise over time (Figure 2G), demonstrating that EBIV can spread from the periphery to the central nervous system from 2 d.p.i.

### EBIV Causes Prominent Histopathologic Changes in the Periphery of BALB/c Mice

**Figure 3:**
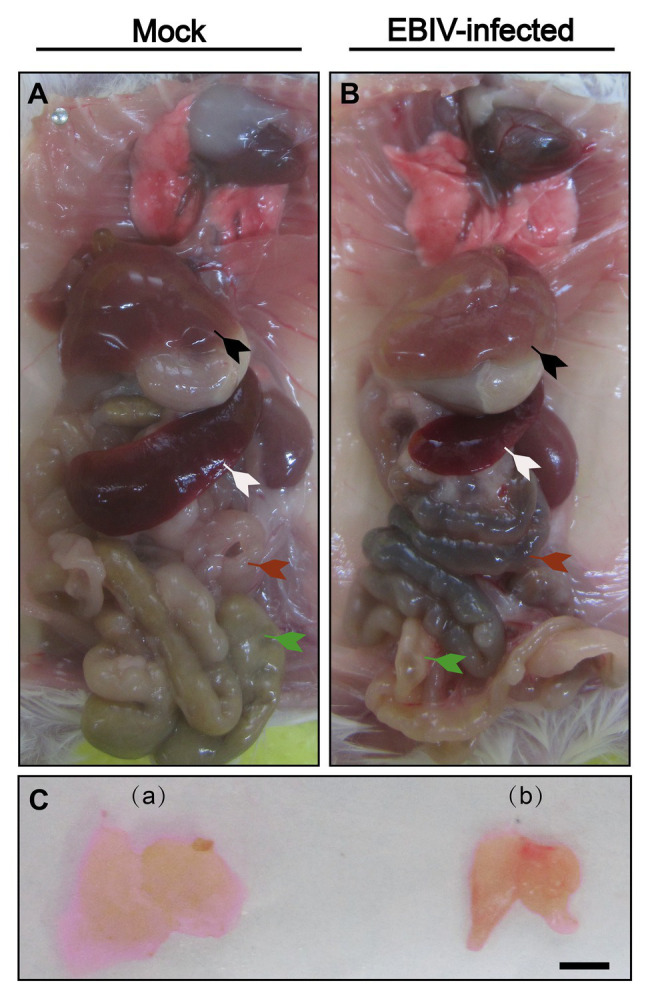
Gross examination of organs in female BALB/c mice following EBIV infection. Representative pictures of organs in mock-infected mice **(A)**+**(C-a)** and EBIV-infected mice **(B)**+**(C-b)**. Appearance of liver color of EBIV-infected mice lighter brown compared to that of mock-infected mice (black arrow). Spleen (white arrow) and thymus (a,b) of EBIV-infected mice achieved a significant reduction in size compared to those of mock-infected mice. The intestine was almost empty (green arrow), and accompanied by severe congestion (red arrow).

The color of the liver from EBIV-infected mice was faded compared with the mock-infected group (Figure 3, black arrow). Additionally, the size of spleen (peripheral immune organ; Figure 3, white arrow) and thymus (central lymphoid organ; Figure 3C) from EBIV-infected mice became smaller, suggesting EBIV infection may cause damage to both central and peripheral immune systems. It was hard to observe food as severe congestion (Figure 3, red arrow) happened in the intestine of infected mice (Figure 3, green arrow), possibly caused by decreased appetite of the mice on 2 d.p.i (Figure 3, red arrow).

### Histopathology and Immunohistochemical Staining Showed Tissue Damage Following EBIV Infection

**Figure 4:**
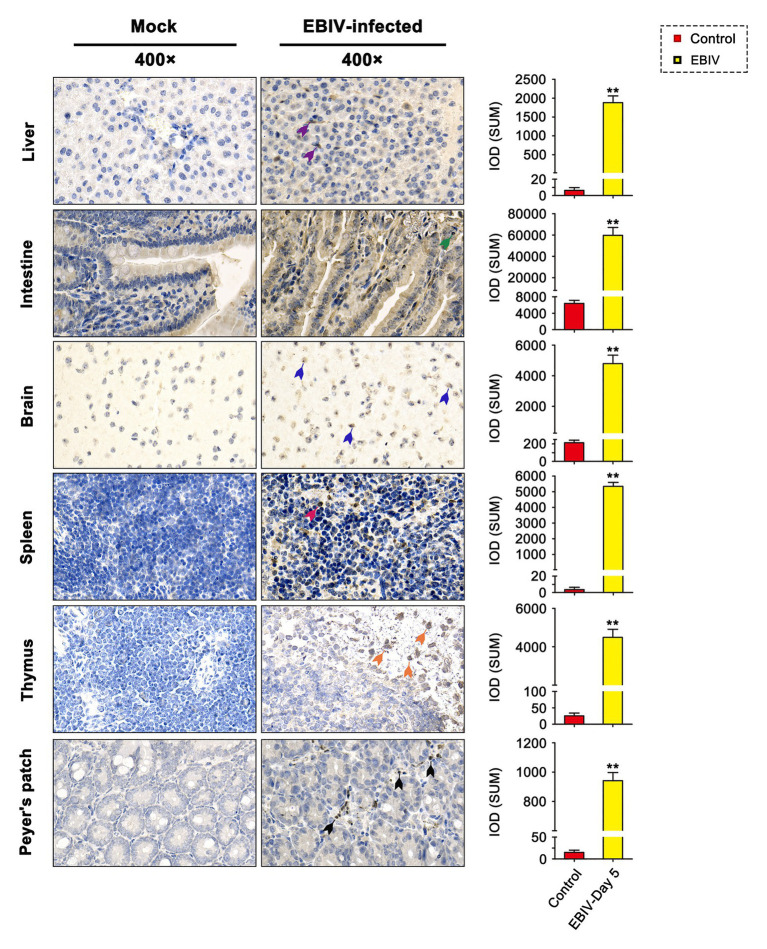
Immunohistochemical (IHC) findings in female BALB/c mice following EBIV-infection at 5 days post-infection (d.p.i). Original magnification was 400× in tissues. Infected animals exhibited positive immunostaining (brown) for EBIV with increased significant IOD differences in the Liver, intestine, brain, spleen, thymus, and Peyer’s patch sections. Viral antigen can be found scattered in Kupffer cells of liver sections (purple arrow), gliocytes of brain sections (blue arrow), and neutrophils of the intestine (green arrow), spleen (red arrow), thymus (orange arrow), and Peyer’s patch (black arrow) sections. Significance was determined by comparing to the control. Error bars represent SD. The two-tailed *p* values are indicated as follows: ^**^p ≤ 0.01.

**Figure 5:**
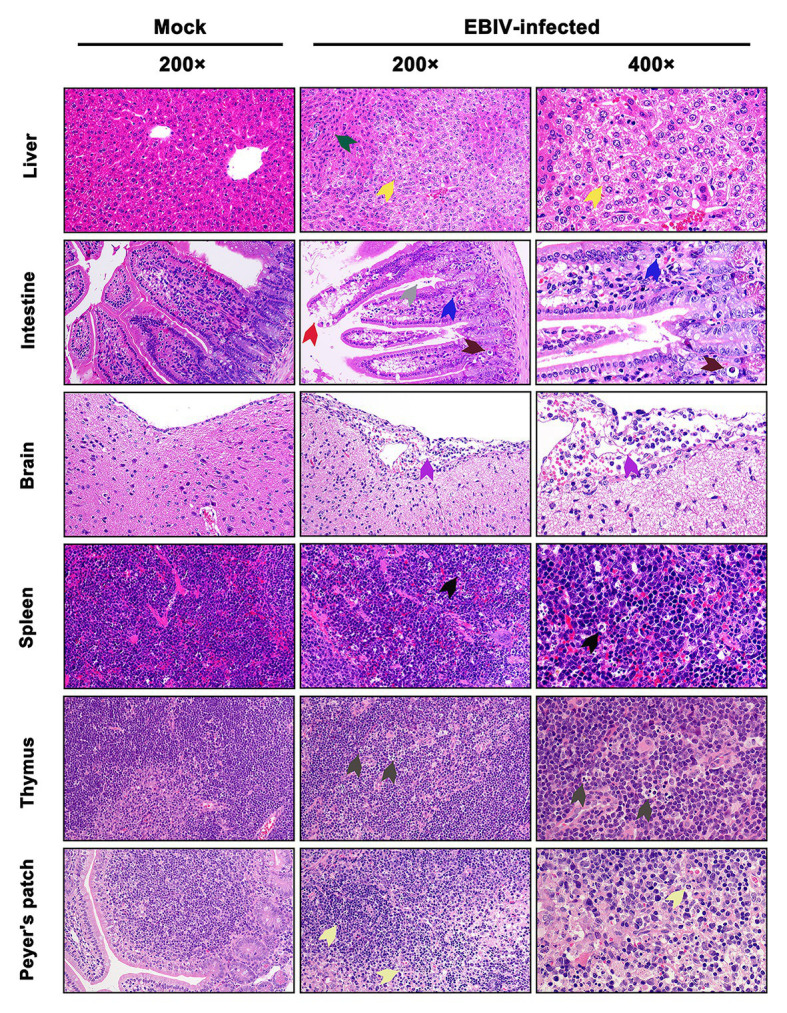
Histopathologic examination in EBIV-infected female BALB/c mice by i.p route at 5 d.p.i. Original magnification was 200× or 400×. Liver: showed hepatocellular edema (yellow arrow) and disordered arrangement (green arrow). Intestine: showed damaged structure (gray arrow) and exfoliated epithelial cells (red arrow) in the intestinal villi, increased neutrophils (blue arrow), and cell necrosis (brown arrow) in the mucosal layer. Brain: showed inflammatory cells, including neutrophils, monocytes, macrophages, and lymphocytes, infiltrated around the meningeal blood vessels (purple arrow). Spleen: showed lymphocyte necrosis (nuclear fragmentation; black arrow). Thymus: showed focal necrosis of lymphocytes (dark gray arrow). Peyer’s patch: showed interstitial edema, loosely arranged cells, and lymphocyte necrosis (light yellow arrow).

At 5 d.p.i, viral antigen was detected in multiple tissues, including liver, intestine, brain, spleen, thymus, and Peyer’s patch by IHC staining. Within the liver of EBIV infected mice, viral antigen showed scattered granular staining (brown) in foci of occasional Kupffer cells (Figure 4, purple arrow). We also observed some disorganized hepatocytes (Figure 5, green arrow) and many hepatocytes of the liver parenchyma exhibited swelling and their cytoplasms loose (Figure 5, yellow arrow). For the intestine of EBIV infected mice, the necrotic mucosal had increased neutrophils (Figure 5, blue and brown arrows), in which viral antigen (Figure 4, green arrow) was detected. The structure of intestinal villi was partially damaged following EBIV infection (Figure 5, gray arrow), accompanied by the shedding of epithelial cells (Figure 5, red arrow). In the brain, we observed viral antigen in gliocytes (Figure 4, blue arrow). A large number of inflammatory cells, including neutrophils, monocytes, macrophages, and lymphocytes, infiltrated around the meningeal blood vessels, which may be associated with mild meningitis (Figure 5, purple arrow). Additionally, viral antigen was largely detected in neutrophils of lymphoid organs, which exhibited severe damage following EBIV infection (Figure 4, red, blue, and black arrows). Lymphocytes underwent necrosis accompanied by nuclear fragmentation in spleen (Figure 5, black arrow). We also observed the irregular structure of thymocytes, with lymphocytes degeneration and focal necrosis in the cortex of thymus for EBIV-infected group. The same histopathology changes were shown in the Peyer’s patch of EBIV infected group (Figure 5, light yellow arrow). Generally, EBIV could infect both the periphery and CNS, causing histopathology changes.

### EBIV Causes Lesions in Liver, Brain, and Spleen

**Figure 6:**
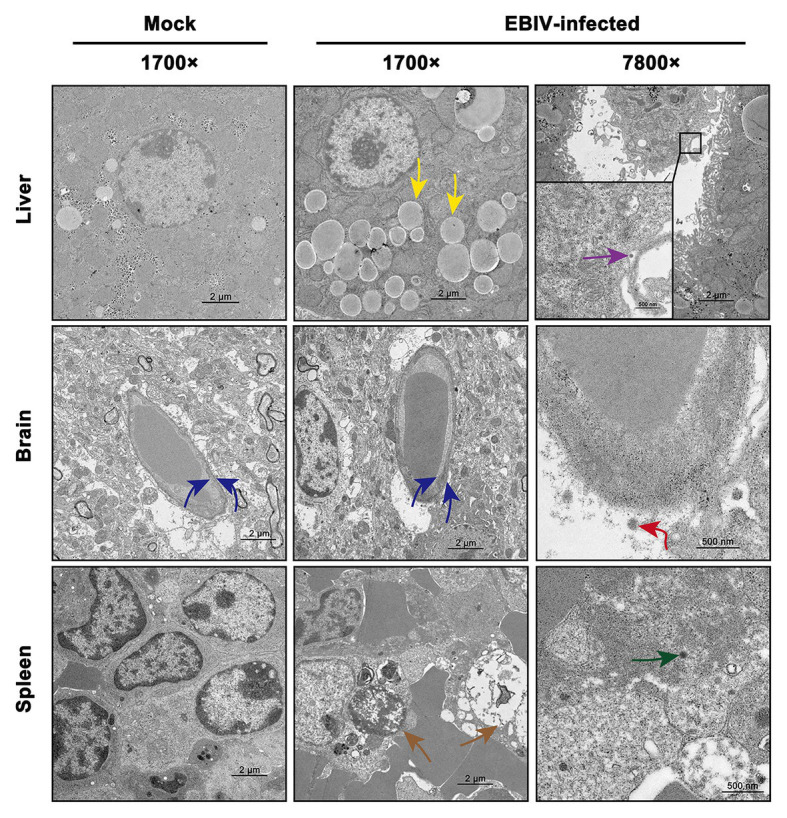
Transmission electron microscopy analysis of tissues infected with 10 PFU of EBIV i.p at 5 d.p.i in female mice. Original magnification was 1700× or 7800×. Liver: lipid droplets in some hepatocytes increased (yellow arrow) and viral particles were found in the Kupffer cell of liver sinus. Brain: the capillary endothelial cells (BCECs) swelled (blue arrow) and viral particles were detected (red arrow). Spleen: lymphocytes were necrotic (brown arrow) and viral particles were seen in the granulocyte (green arrow).

We also observed the ultrastructural characteristic of tissues of EBIV-infected BALB/c mice at 5 d.p.i. Viral particles were located in Kupffer cells of the liver (Figure 6, purple arrow), which was consistent with the detection of viral pathogens in Kupffer cells, as mentioned previously (Figure 5, purple arrow). Lipid drops increased significantly in the liver (Figure 6, yellow arrow) of the infected group, resulting in hepatic steatosis. In CNS, viral particles were observed in the cerebral cortex of the brain (Figure 6, red arrow), which lead to the destruction of the endothelial wall and edema (Figure 6, blue arrow). Consistent with previous HE and IHC staining of the spleen, massive lymphocytes were necrotic (Figure 6, brown arrow), and virus particles could be observed in these damaged granulocytes (Figure 6, green arrow).

### EBIV Infection Causes Abnormal Blood Constituents in BALB/c Mice

**Figure 7:**
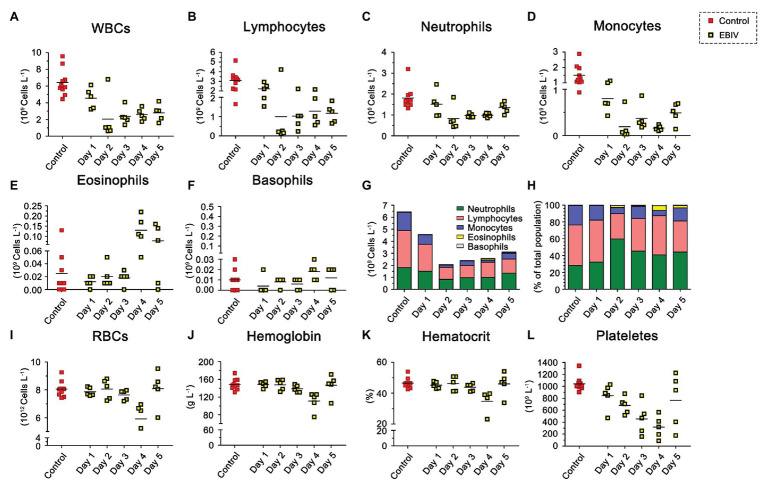
Hematologic abnormalities induced by EBIV infection with 10 PFU in female mice. BALB/c mice were inoculated by i.p route (*n* = 5) and 10 mock-infected animals as control, Each symbol represents one animal. **(A)** Total White blood cells (WBCs). **(B)** Lymphocyte. **(C)** Neutrophil. **(D)** Monocyte. **(E)** Eosinophil. **(F)** Basophil. Composite graph showing average absolute counts **(G)** and average population proportions **(H)** of lymphocytes, neutrophils, monocytes, eosinophils, and basophils. **(I)** Total red blood cells (RBCs). **(J)** Hemoglobin (Hb). **(K)** Hematocrit. **(L)** Platelets.

To evaluate the hematological and clinical chemistry parameters, we analyzed the WBC. The proportion of lymphocytes decreased at 2 d.p.i, while the proportion of eosinophils increased at 4 d.p.i (Figure 7H). However, the total WBC counts in infected mice started to decrease from 1 d.p.i and then showed a significant drop at 2 d.p.i, accompanied by a mild recovery in the following days that could not reach the original level (Figures 7A,G). Further analysis showed that the counts of lymphocytes, neutrophils, and monocytes were consistent with the trend of overall lymphocytes (Figures 7B–D). Eosinophils continued to increase throughout the study, especially from 4 d.p.i (Figure 7E). We did not observe a noticeable change in basophil counts between the two groups (Figure 7F). Compared to the mock group, infected mice had decreased RBC counts (Figure 7I), especially platelet numbers, which decreased evidently (Figure 7L), which might be due to their migration to infection sites. Levels of Hb (Figure 7J), and hematocrit (Figure 7K) also decreased at 4 d.p.i but were partially restored at 5 d.p.i. To further understand the physiological status of EBIV infection in mice, we measured the clinical chemistry of serum. The ALT and LDH-L were significantly elevated, suggesting that the liver was damaged following EBIV infection. The level of CK increased significantly, which indicated a minor brain injury. Only GLU level decreased, which might be caused by mice decreased appetite (Supplementary Figure S1).

### EBIV Infection Induce Cytokines Change in Target Organs

**Figure 8:**
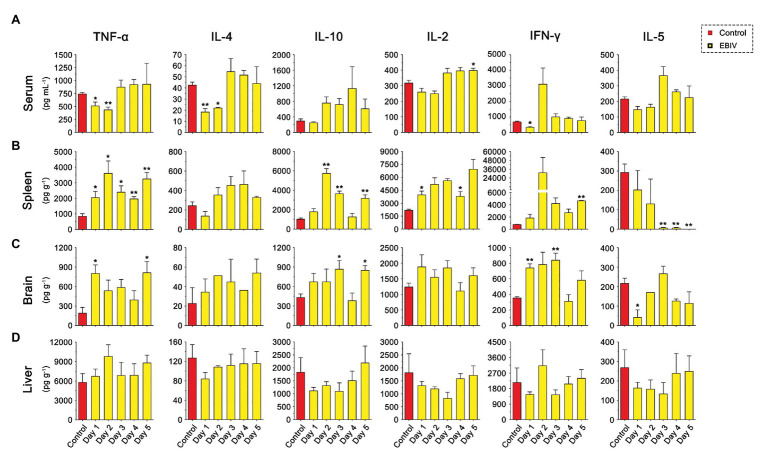
Cytokine abnormalities induced by EBIV infection in female mice. **(A)** IL-2, IL-4, IFN-γ, TNF-α, IL-10, and IL-5 levels were determined in serum **(A)** and in different tissues, spleen **(B)**, brain **(C)**, and liver **(D)** of mock and EBIV-infected mice. All cytokine concentrations in tissues were normalized to the mass of the respective homogenized tissue. Significance was determined by comparing to the control. Error bars represent SD. The two-tailed *p* values are indicated as follows: ^*^p ≤ 0.05; ^**^p ≤ 0.01.

As shown in Figure 8, the inflammatory related cytokines, TNFα and IL-4 levels were significantly decreased in the serum at the earlier stage of EBIV infection, but recovered to the normal level at 3 d.p.i. However, we found a rising trend for TNFα and IL-4 in both spleen and brain of infected mice, especially TNFα level in the spleen were increased markedly from 1 to 5 d.p.i. IL-10 production peaked in the spleen at 2 d.p.i then decreased, but still was significantly higher compared to the control. We also observed a markedly increased brain IL-10 at 3 and 5 d.p.i. These results indicated an inflammation change in the peripheral and CNS. Significantly, an increase in IL-1β levels was observed in spleen at 2 and 3 d.p.i, in liver at 2 d.p.i (Supplementary Figure S2). Surprisingly, no IL-1β levels were detected in the brain. The serum IL-2 exhibited an increasing trend at a later stage of infection, which was significantly high at 5 d.p.i. IL-2 levels in the spleen increased significantly at 1 and 4 d.p.i. IFNγ levels had a sharp decrease at 1 d.p.i in serum and were enhanced in brain at 1 and 3 d.p.i, suggesting EBIV infection altered IFNγ produced innate immune cells, especially NK cell function during the early infection. As a cytokine to mediate B cell differentiation, IL-5 level in the spleen was sharply reduced at the later stage of EBIV-infection, even undetectable on 5 d.p.i, indicating the damage of B cell function caused by EBIV. Interestingly, in the brain, IL-5 was significantly decreased at 1 d.p.i and returned to the similar level as control. There was no obvious difference for these cytokines in liver (Figure 8D) suggesting the complexity of host immune response.

## Discussion

The immunocompetent, 6–8-weeks-old BALB/c mice were used for EBIV infection by intraperitoneal inoculation in this study. This animal model was previously developed as an experimental host of Oropouche virus (OROV) that belongs to Simbu serogroup of *Orthobunyavirus*, with severe involvement of the central nervous system (Santos et al., 2012, 2014). Our results indicated that EBIV could cause acute disease in BALB/c mice. A large majority of infected animals developed disease on the 2 d.p.i, progressing to death within 9 days. However, this very rapid disease progression caused by EBIV is a rare case in *Orthobunyavirus* infection in the adult mice model. In general, weanling (3 weeks old or younger) mice are more susceptible to *Orthobunyavirus* infection, and adults (greater than 6 weeks) may be resistant (Bennett et al., 2011; Santos et al., 2012; Winkler et al., 2017; Zhang et al., 2017). What leads rapidly to disease and death in BALB/c mice? In experimentally infected mice, EBIV might induce encephalopathy with multiple organ damage. EBIV-infected mice developed significant hepatic damage on the 5 d.p.i, confirmed by histopathological studies and changes in the serum levels of ALT/LDH-L, resulting in hepatic disease (Camini et al., 2014). The mammalian peripheral lymphoid organ plays a central role in host defense (Golub et al., 2018). EBIV could replicate in most lymphoid tissues, including spleen, thymus, and Peyer’s patch, resulting in lymphocyte necrosis (Figures 4, 5), and atrophy of spleen and thymus were observed. This is unusual in *Orthobunyavirus* infection (Wernike et al., 2012). The IL-1β is a key mediator of the inflammatory response (Lopez-Castejon and Brough, 2011), which is essential for host immune response. The rising level of IL-1β in spleen and liver demonstrated the occurrence of inflammation in the periphery at the earlier stage of EBIV infection. Besides, IL-5 is initially defined as a key mediator of activated B cell differentiating to antibody-secreted B cells (Randall et al., 1993). Significant abolishment of IL-5 of the spleen at the later infection stage, suggests a possible antibody deficiency. So we performed the neutralization assay to detect neutralizing antibody in serum samples of mice from 1 to 5 d.p.i and found that even undiluted serum of all mice could only reduce plaques by 30% (data not shown). The infiltrating inflammatory cells to the CNS and increase of CK indicated brain damage, and the viral titer in the brain continued to increase following EBIV infection. Viruses may cross the blood-brain barrier (BBB) *via* several routes, including direct infection of BMECs, transcellular or paracellular viral trafficking across the endothelium (Verma et al., 2009; Daniels et al., 2014). We found obvious enhancement of TNF-α and IL-10 in both spleen and brain, indicating an inflammation status of BALB/c following EBIV infection. TNF-α is a key factor for viral crossing of the BBB (Miner and Diamond, 2016), and IL-10 was also proved to facilitate viral infection (Bai et al., 2009). TNF-α and IL-10 may play an important role in EBIV entering the brain (Den Heijer et al., 2010; Razakandrainibe et al., 2013). Thus, the released host factors might (cause by the damage to the periphery like spleen), increase the permeability of the BBB, and make it easier for a virus to invade the CNS either directly or by crossing the BMEC tight junctions (Daniels et al., 2014). These studies indicate that the innate immune response in adults is not sufficient for protection and that components of the adaptive immune response is necessary to prevent the virus from invading the CNS (Winkler et al., 2017).

The transmission of arboviruses to vertebrates through mosquito vectors is an intricate complex process, and establishing vector transmission by bite is the most relevant mode in mimicking arboviral disease infection (Secundino et al., 2017). In a previously conducted study by Pingen and team, they note that mosquito bites could significantly enhance infection with BUNV infection (Pingen et al., 2016). Although EBIV has been isolated from *Culex modestus* (Xia et al., 2020), the primary transmission vector of EBIV is still not clear. And we have not yet detected any EBIV RNA in the sampled *Aedes flavipectus*, *Aedes Caspian*, and *Culex pipiens* mosquitoes that formed part of our mosquito surveillance studies for the years 2014 and 2019 (data not shown). Notably though, in the same serogroup of Bunyamwera, experimental studies have shown that *Ae. aegypti* is a competent vector in transmission of BUNV, and despite the Ngari virus (NRIV) being isolated from many mosquito vectors, it is only *Anopheles gambiae Giles* that has proved to be competent vector for NRIV (Dutuze et al., 2018). Therefore, the long-term surveillance of mosquitoes and experimental infection EBIV in different mosquito species should be conducted to investigate the vector competence for EBIV, as well as its tropism in the positively identified competent mosquito vectors.

The reported hosts of Bunyamwera serogroup viruses are rodents, sheep, cattle, equine, avian species, primates, or humans (Dutuze et al., 2018). Being a not well-characterized virus, we do not know whether EBIV can cause disease to humans even though the seroprevalence clue of EBIV infection in humans has been reported (Xia et al., 2020). It is possible that other vertebrate species, such as small mammals or birds could be more susceptible to EBIV. In order to investigate the possible infection of EBIV in avian species, we conducted an experimental infection of EBIV in the embryonated chicken egg (ECE). Our obtained findings showed that EBIV can infect 6-days old ECE by yolk sac route of inoculation and cause 100% death at titers of 10^6^ PFU/egg. Thus, these findings indicate that EBIV can infect the avian species (data not shown). Unfortunately, the detection of EBIV antibodies in wild animals or birds has not been done so far. Overall, the comprehensive study for these lesser-characterized Bunyaviruses is critical to our best preparation for future threats.

## Data Availability Statement

The original contributions presented in the study are included in the article/Supplementary Material; further inquiries can be directed to the corresponding authors.

## Ethics Statement

The animal study was reviewed and approved by Laboratory Animal Ethics Committee of Wuhan Institute of Virology, Chinese Academy of Sciences.

## Author Contributions

HX and ZY designed the experiments. LZ, DH, PY, QD, and CM performed the experiments. LZ, HL, PY, and HY analyzed the data. LZ and JY contributed the reagents, materials, and analysis tools. LZ, HL, EA, RN, GZ, DB, ZY, and HX wrote and review the manuscript. All authors contributed to the article and approved the submitted version.

### Conflict of Interest

GZ was employed by the company Xinjiang Heribase Biotechnology Co., Ltd.

The remaining authors declare that the research was conducted in the absence of any commercial or financial relationships that could be construed as a potential conflict of interest.
