# Supplementary material for: Pathogenesis and Immune Response of Ebinur Lake Virus: A Newly Identified Orthobunyavirus That Exhibited Strong Virulence in Mice
Source: Front Microbiol. 2021 Feb 1;11:625661. doi: 10.3389/fmicb.2020.625661 (PMC7882632; doi:10.3389/fmicb.2020.625661)
Supplement: Supplementary file 1 [file Data_Sheet_1.doc]

**
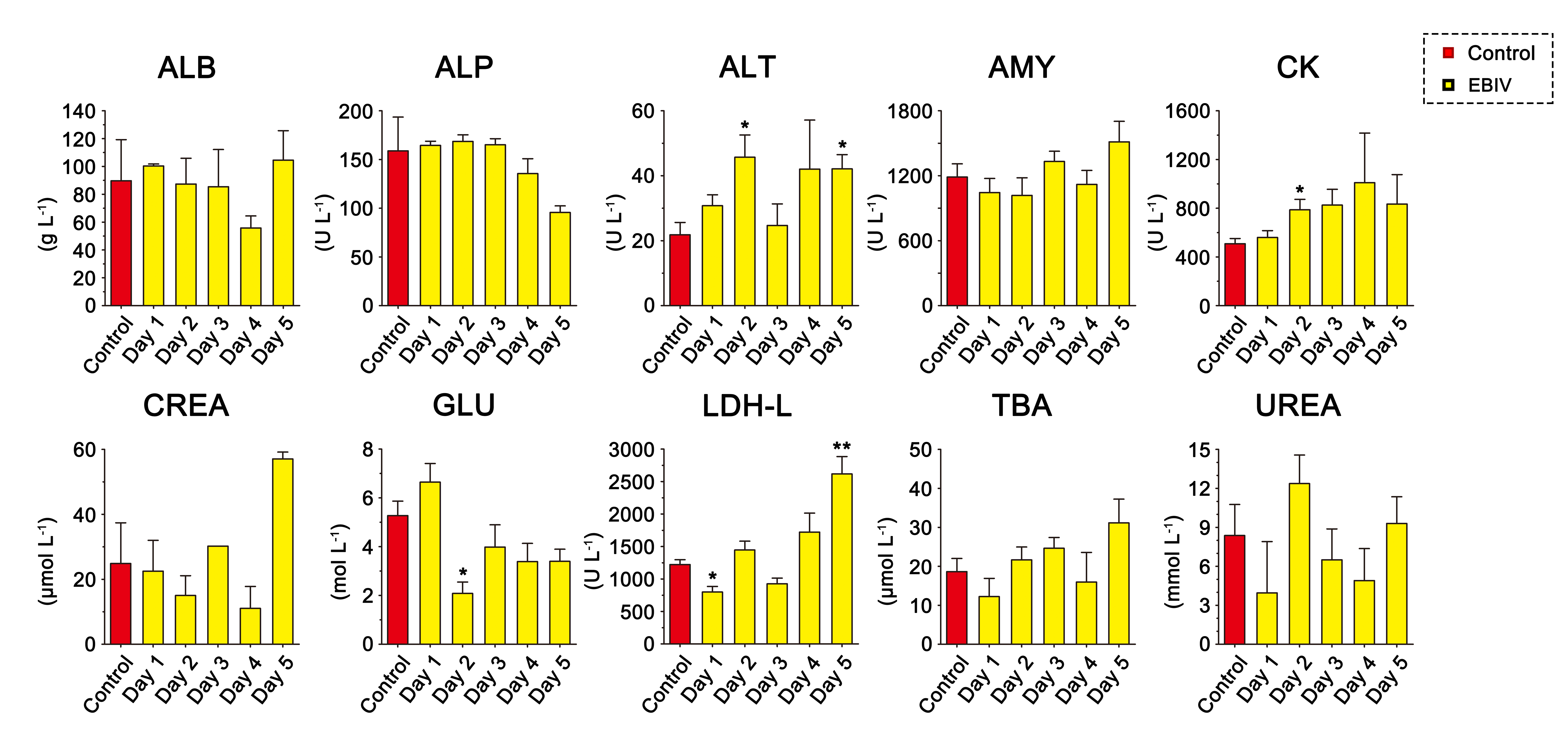
**

**Supplementary Figure 1. Clinical chemistry abnormalities induced by EBIV infection.** Results for ALB, ALP, ALT, AMY, CK, CREA, GLU, LDH-L, TBA, and UREA were shown for the serum. Significance was determined by comparing to the control. Error bars represent standard deviations. The two-tailed P values are indicated as follows: *, P ≤ 0.05; **, P ≤ 0.01


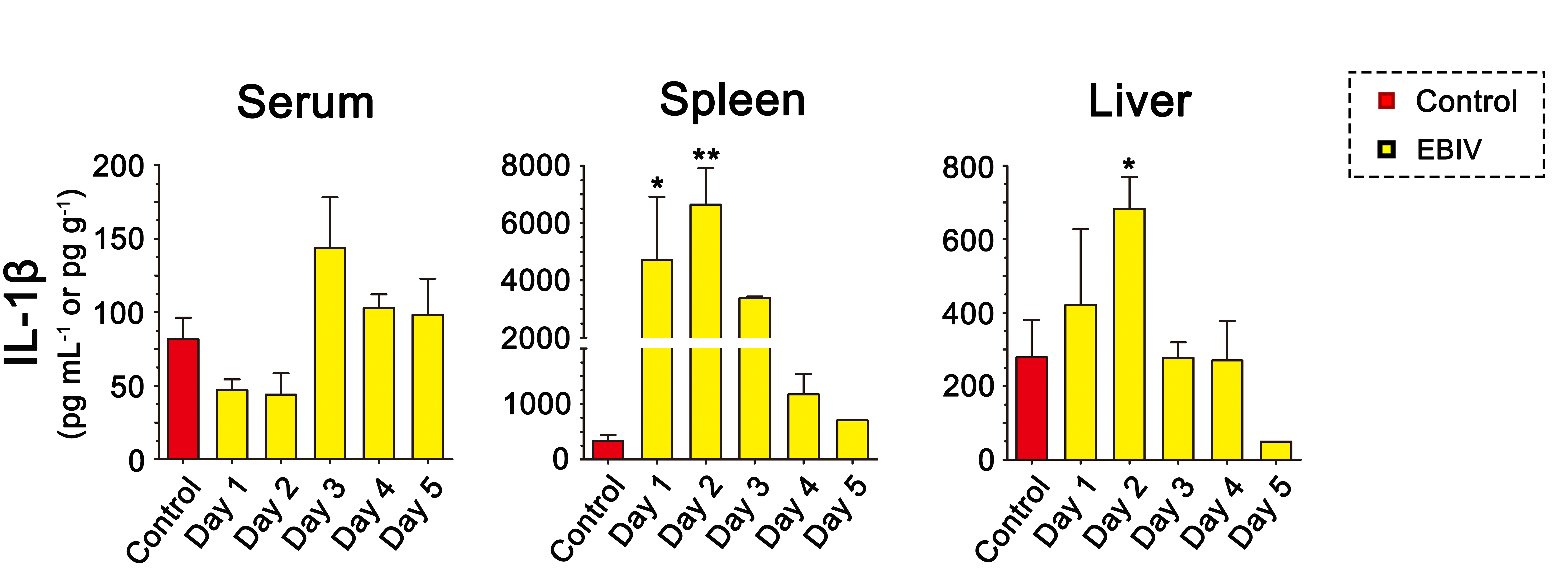


**Supplementary Figure 2.** **IL-1β levels were determined in serum and, spleen, and liver of mock and EBIV-infected female mice.** All cytokine concentrations in tissues were normalized to the mass of the respective homogenized tissue. Significance was determined by comparing to the control. Error bars represent standard deviations. The two-tailed P values are indicated as follows: *, P ≤ 0.05; **, P ≤ 0.01.
